# Hypothesis of snake and insect venoms against Human Immunodeficiency Virus: a review

**DOI:** 10.1186/1742-6405-6-25

**Published:** 2009-11-19

**Authors:** Ramachandran Meenakshisundaram, Shah Sweni, Ponniah Thirumalaikolundusubramanian

**Affiliations:** 1 Madras Medical College, Chennai, India; 2 University of Debrecen, Medical & Health Science Center, Debrecen, Hungary; 3 Chennai Medical College Hospital & Research Center, Irungalur, Trichy, India; 4 1103, Dimple Heights, Asha Nagar, Kandivali East, Mumbai - 400101, India; 5 Simonyi utca, 35, fldsz 30, Debrecen 4028, Hungary

## Abstract

**Background:**

Snake and insect venoms have been demonstrated to have beneficial effects in the treatment of certain diseases including drug resistant human immunodeficiency virus (HIV) infection. We evaluated and hypothesized the probable mechanisms of venoms against HIV.

**Methods:**

Previous literatures published over a period of 30 years (1979-2009) were searched using the key words snake venom, insect venom, mechanisms and HIV. Mechanisms were identified and discussed.

**Results & Conclusion:**

With reference to mechanisms of action, properties and components of snake venom such as sequence homology and enzymes (protease or L- amino acid oxidase) may have an effect on membrane protein and/or act against HIV at multiple levels or cells carrying HIV virus resulting in enhanced effect of anti-retroviral therapy (ART). This may cause a decrease in viral load and improvement in clinical as well as immunological status. Insect venom and human Phospholipase A_2 _(PLA_2_) have potential anti-viral activity through inhibition of virion entry into the cells. However, all these require further evaluation in order to establish its role against HIV as an independent one or as a supplement.

## Background

Components of snake venom are used for health and diseases[1], an interesting emerging concept. Some of the snake venom preparations include angiotensin-converting enzyme (ACE) inhibitor, disintegrins (antiplatelet aggregants)[2] and also used, in diagnostic assays of various blood coagulation factors[3]. Alpha neurotoxin, extracted from cobras has been shown to have analgesic effects [4,5] and crotoxin from *Crotalus durissus terrificus *has cytotoxic effects[6]. Recently, Alrajhi and Almohaizeie[7] demonstrated the usefulness of snake venom in a patient suffering from a drug resistant human immunodeficiency virus (HIV) infection, who was on anti-retroviral therapy (ART). In HIV patients, the response after administration of snake venom preparation [7,8] was an increase in CD4 count and decrease in viral load. We have recently shown that the components of snake venom might enhance the activity of ART at different levels[9]. Interestingly, insect venom and human secretions also have anti-HIV activity [10-12]. Hence, we evaluated and hypothesized the probable mechanisms of venoms and secretions against HIV infection.

## Methods

Previous literatures published over a period of 30 years (1979-2009) were searched using the key words snake venom, insect venom, HIV and mechanisms. Based on the available materials, the probable mechanisms of action of venom and secretions against HIV were identified and discussed.

## Results and Discussion

### Snake Venom

The pharmacological activities of snake venom are complex in nature with little known about them and it varies amongst the multitude of snake venoms. The mechanisms of action of snake venom against HIV are mediated through various levels [9], such as structural homology, binding interference (receptor/enzyme), catalytic/inhibitory activity through enzymes, and induction/interaction at membrane level.

#### 1) Structure

The HIV virus entry into cells is mediated through the binding of envelope glycoprotein - gp120 [13]. There is a striking homology between the sequence 164-174 of short segment HIV-1 gp120 and the highly conserved 30-40 amino acid residues of snake venom neurotoxins long loop [14,15]. Thus, both may compete for the same receptor or binding site and act against HIV.

F N I S T S I R G K V - HIV gp 120

C D K F C S I R G P V - alpha - cobratoxin *(Naja naja siamensis)*

C D A F C S I R G K R - k - bungarotoxin (*Bungarus multicintus)*

Structure 1: Amino acid sequences of HIV gp120 (164-174) compared to alpha- cobratoxin and k- bungarotoxin (30-40)[15].

#### 2) Binding

a) Snake venom contains Phospholipase A_2 _(PLA_2_)[11,16], which protect human primary blood leukocytes from the replication of various macrophage and T cell-tropic human immunodeficiency virus 1 (HIV-1) strains. PLA_2 _which is found in the venom of many snakes has been shown to block viral entry into cells before virion uncoating through prevention of intracellular release of viral capsid protein [16]. This is mainly due to the specific interaction of PLA_2 _to host cells and not due to catalytic activity.

b) Immunokine - an oxidized derivative of alpha - cobra toxin (*Naja naja siamensis*), has been shown to inhibit the infection of lymphocytes by HIV and Feline immunodeficiency virus (FIV) through chemokine receptors (CCR 5 and CXCR 4) [17].

#### 3) Enzymatic activity

a) L- amino acid oxidase (LAO), present in the venom of *Trimeresurus stejnegeri*[18], *C. Atrox, P. australis*[19]; inhibits infection and replication of HIV virus through P24 antigen in a dose dependant manner[18]. P24 antigen is a core protein of HIV and its level associates with viral load[20]. Besides the binding of protein to cell membrane, hydrogen peroxide (H2O2) produced as a free radical could inhibit the infection/replication of HIV, thereby further enhancing the anti viral activity. In contrast, catalase - a scavenger of H2O2, reduces the anti- viral activity [18].

b) Protein fragment isolated from *Oxyuranus scutellatus *snake venom is a potent inhibitor of p24 antigen and blocks viral replication of resistant strains [21].

c) Snake venom contains metalloprotease inhibitors[16,22] which could prevent the production of new viruses through inhibition of protease enzymes. HIV infects a CD4 cell of a person's body and then it copies its own genetic code into the cell's DNA. Then, CD4 cell is "programmed" to make new HIV genetic material and proteins. These proteins are degraded by HIV protease enzyme and again these proteins are used to make functional new HIV particles. Protease inhibitors are used to block the protease enzyme and prevent the cell from producing new viruses.

#### 4) Effect on membrane protein

P-glycoprotein (P-gp), a membrane protein, is an energy-dependent efflux transporter driven by ATP hydrolysis[23]. P-gp transports a wide range of substances with diverse chemical structures. In general, P-gp substrates appear to be lipophilic and amphiphatic, and are recognized to play an important role in processes of absorption, distribution, metabolism, and excretion of many clinically important drugs in humans [23]. Because of its importance in pharmacokinetics, inhibition or induction of P-gp by various components of snake venom can lead to significant drug-drug interactions, thereby changing the systemic or target tissue exposure of the protease inhibitors. At the same time one has to remember genetic polymorphism of P-gp,[23] which has also been recorded recently, because it may affect drug disposition and produce variable drug effects.

### Other Clinical Uses of Snake Venom

Neurotoxins from snake such as cobra venom activates central cholinergic pathways by nicotine and nicotinic agonists, which have been shown to elicit anti-nociceptive effects in a variety of species and produces significant analgesic effect [24,25]. PLA_2 _inhibitors (PLI) from snake - *Habu snake, Trimeresurus flavoridis *have anti-enzymatic, anti-myotoxic, anti-edema inducing, anti-cytotoxic, and anti-bacterial activities - [26], and hence, used in neurodegenerative disorders such as trauma, Alzhiemers disease, Parkinson's and brain tumors - [27]. Fibrolase from *A. contorix *snake venom degrade α and β chains of fibrin and used as a thrombolytic agent [28]. Snake venom RGD-disintegrins showed direct interaction in several tumor cell lines. It blocks αvβ3 integrin in tumor cells, thus inhibited their adhesion to the extra cellular matrix and thereby prevents metastasis [29]. PLA_2 _from *Bothrops neweidii and Naja Naja venom*, was found to be cytotoxic towards B16F10 melanoma and Ehrlich ascitic tumor cells, as an anti-cancer drug [30]. Crotoxin, a pre-synaptic neurotoxin has been tried as an anti-cancer agent in advanced cancer patients [31]. VRCTC-310, a natural product with PLA_2 _from *Crotalus Durissus terrificus *and cardiotoxin from *Naja Naja atra*, have inhibitory effect against human and murine tumor cell lines, and have effective value in the treatment of advanced solid cancers, which were refractory to other therapy [32].

### Insect Venom

#### 1. Gene expression

Melittin is a 26 amino acid amphipathic α-helical peptide, a major component of bee venom [33]. The cecropins are a family of antibacterial peptides 35-39 amino acids in length which occur in a number of insect species and in mammals [34]. Like melittin, they consist of two α-helices linked by a flexible segment, and contain amphipathic structures. Melittin and cecropin act against a wide range of infectious agents, including Gram-positive and Gram-negative bacteria [35]. Whereas melittin is lytic for red blood cells at high concentrations, cecropins do not lyse erythrocytes or other eukaryotic cells [35] and appear to be non-toxic for mammalian cells. Melittin has been reported to inhibit replication of murine retroviruses, tobacco mosaic virus [36] and herpes simplex virus [37] suggesting that melittin also displays antiviral activity. Analogous to antibacterial activity, the antiviral activity of melittin has been attributed to direct lysis of viral membranes, as demonstrated for murine retroviruses [38]. However, melittin also displays antiviral activity at much lower, non-virolytic concentrations, as shown for T cells chronically infected with HIV-1 [39]. Wachinger [10] et al., reported that melittin and cecropin A are shown to suppress production of HIV-1 by acutely infected cells and also, suppresses the HIV-1 replication by interfering with host cell-directed viral gene expression [10]. Melittin treatment of T cells reduces levels of intracellular Gag and viral mRNAs, and decreases HIV long terminal repeat (LTR) activity. Besides, HIV LTR activity is also reduced in human cells stably transfected with melittin and cecropin genes.

#### 2. Binding

i. Mammalian venom secreted PLA_2 _have been associated with a variety of biological effects. Fernard et al [11] suggested that PLA_2 _protect human blood leukocytes from the replication of various macrophage and T cell-tropic HIV-1 strains. This is neither due to virucidal nor cytotoxic effect on host cells; however PLA_2 _blocks viral entry into cells before virion uncoating, independent of the receptor. Inhibitors and catalytic products of PLA_2 _have no effect on HIV-1 infection suggesting that PLA_2 _catalytic activity is not involved in antiviral effect.

ii. Peptide p3bv, is a 21-25 aminoacids component from secreted phospholipases of bee venom (bvPLA_2_) [40]. The p3bv peptide inhibits the replication of HIV-1 through prevention of the cell fusion process mediated by T-lymphotropic HIV-1 envelope without the effect of monocytotropic HIV-1. Then, p3bv inhibits the binding of stromal cell factor-1 α (natural ligand of CXCR4) and 12G5 (anti-CXCR4 monoclonal antibody). Overall, p3bv blocks the replication of T-lymphotropic HIV-1 strains by interacting with CXCR4, thereby blocking viral entry into cells.

iii. PLA_2_-I A from bee, and serpent venom showed in vitro anti-HIV activity, which was due to the ability of secretions to destabilize anchorage (heparans) and fusion (cholesterol) receptors on HIV target cells [41].

### Human PLA_2_

Interestingly, human PLA_2 _(group III PLA_2_) has significant homology with bee venom PLA_2 _[42]. Several murine and human group phospholipases such as II A, X, V, XII, II E, I B, and II F have potential antibacterial effects against gram positive and negative bacteria [43]. In individuals repeatedly exposed to HIV but who remain uninfected, several possible reasons for protection have been proposed but not clearly elucidated [44].

#### 1. Membrane

Kim et al., [12] suggested that human PLA_2 _and human group X PLA_2 _(PLA_2_-X) have potential antiviral activity against diverse lentiviruses by the degradation of viral membrane. PLA_2_-X has high affinity for phosphatidylcholine, a phospholipid in outer plasma membrane and hydrolyzes it. Viral membrane of HIV- 1 is rich in phosphatidylcholine and sphingomyelin and may be more susceptible to PLA_2_-X.

#### 2. Binding

PLA_2_-X inhibits replication of both CXCR4 and CCR5 HIV-1 in human CD4 cells. This effect was observed despite the resistance of viral preparations to lysis by antibody-mediated complement activation, suggesting that this action occur in cases even where the acquired immunity is ineffective[12]. In view of the above, anitiviral activity of human PLA_2 _expressed in immune tissues and cells will be particularly interesting to analyze in future [44].

### Debate in PLA_2 _action

Kim et al., [12] concluded that enzymatic activity of PLA_2_-X is necessary for antiviral effect, which contradict the findings of Fernard et al., [11] where catalytic activity was not required. Hence, further studies are needed to ascertain its exact mechanism.

## Conclusion

In view of the above mechanisms, snake venom might reduce HIV load, thereby decreasing its effect and enhances CD4 count. Insect venom and human PLA_2 _act through PLA_2 _mediated inhibition of virion entry into host cells. Hopefully, the use of venom preparation or a synthetic molecule similar to snake/insect venom/human secretions without adverse effects may open a new era of anti-retroviral therapy against HIV or act as an adjuvant not only for HIV but also to other viral infections. However, further research is required to ascertain the exact mechanism of antiviral activity of snake and insect venoms.

## List of abbreviations

HIV: human immunodeficiency virus; ART: anti-retroviral therapy; PLA_2_: Phospholipase A_2_; HIV-1: human immunodeficiency virus 1; ACE: angiotensin-converting enzyme; FIV: Feline immunodeficiency virus; LAO: L- amino acid oxidase; H2O2: hydrogen peroxide; P-gp: P-glycoprotein; PLI: PLA_2 _inhibitors; LTR: long terminal repeat; bvPLA_2_: phospholipases of bee venom; PLA_2_-X: human group X PLA_2_.

## Competing interests

The authors declare that they have no competing interests.

## Financial disclosure

Nil

## Authors' contributions

RM, SS and PT hypothesized and collected references. RM and SS drafted the first version. PT critically revised the manuscript. All authors read and approved the final version.
